# The Significance of an Enhanced Concept of the Organism for Medicine

**DOI:** 10.1155/2016/1587652

**Published:** 2016-06-29

**Authors:** Bernd Rosslenbroich

**Affiliations:** Institute of Evolutionary Biology and Morphology, Centre for Biomedical Education and Research, Faculty of Health, School of Medicine, Witten/Herdecke University, Stockumer Strasse 10, 58453 Witten, Germany

## Abstract

Recent developments in evolutionary biology, comparative embryology, and systems biology suggest the necessity of a conceptual shift in the way we think about organisms. It is becoming increasingly evident that molecular and genetic processes are subject to extremely refined regulation and control by the cell and the organism, so that it becomes hard to define single molecular functions or certain genes as primary causes of specific processes. Rather, the molecular level is integrated into highly regulated networks within the respective systems. This has consequences for medical research in general, especially for the basic concept of personalized medicine or precision medicine. Here an integrative systems concept is proposed that describes the organism as a multilevel, highly flexible, adaptable, and, in this sense, autonomous basis for a human individual. The hypothesis is developed that these properties of the organism, gained from scientific observation, will gradually make it necessary to rethink the conceptual framework of physiology and pathophysiology in medicine.

## 1. Introduction

Modern biology is rich in empirical knowledge about the different levels of life processes, ranging from ecosystems across species and individual organisms down to the structures and functions of cells and subcellular processes. There is, however, a strong tendency to explain more complex entities such as organisms, cells, or proteins by the properties of their individual component parts. Especially in biomedical research, it is more or less tacitly expected that organisms will be explainable one day by the sum of all molecular functions within and around the cell and that they are all controlled by genetic information within the nucleus. Finally, genes are thought to determine all processes of an organism. It became common to view life as optimized for genes rather than for organisms. The view of genes as “selfish replicators,” and phenotypes as perishable vehicles [1], emerged as compelling in both the academic community and the public.

This assumption has been powerful during the last decades, and consequently research in molecular and genetic functions produced vast knowledge and valuable insights. This premise also became very strong in medical science. When the genome is seen as the prime mover, it can thus be expected to find the clues here to many or most of the diseases that plague humans. This was the main driving force for the human genome project and still is a main motivation for biomedical research.

Personalized medicine in particular (more recently often called precision medicine), which at present is becoming a popular area of research, focusses to a large extent on this view. Many authors expect that it will transform medicine altogether. It is the general idea that, by reading the genome or other molecular components of an individual human, it will be possible to predict which diseases or medical conditions the patient might ultimately suffer from. Furthermore, with such knowledge it should then be possible to either prevent the occurrence of the disease or to provide more targeted therapy and improve therapeutic outcomes [2–8].

“Nature glossary,” for example, defines personalized medicine as “the use of genetic susceptibility or pharmacogenetic testing to tailor an individual's preventive care or drug therapy.” Daack-Hirsch and Campbell [9, p. 1] describe personalized medicine as “the ability to customize medicine using molecular information to more accurately understand disease patterns and diagnose disease and to tailor preventive and therapeutic intervention more effectively with fewer side effects,” and Morse and Kim [10, p. 1] write that “personalized medicine is an emerging field with a goal of applying genomic information as a predictor of disease risk as well as individualization of drug therapy.”

Although definitions of personalized medicine differ to a considerable extent and have also changed within the last 10 years [11, 12], this view remains dominant. According to Guttmacher and Collins [13], “genomics-based knowledge and tools promise the ability to approach each patient as the biological individual he or she is, thereby radically changing our paradigms and improving efficacy.”

The perspective is, finally, to detect the “real causes” of diseases rather than curing symptoms. Environmental influences and life history events may modify these conditions, but the origins of problems are predominantly sought at the lower levels of biological organization.

## 2. Inconsistencies of the Biomedical Model

To become more attuned to each individual patient is an ideal of many medical disciplines. There are also often problems with individual reactions, susceptibilities, and health situations in standard medications. Thus, it seems to be tempting to be able to administer more individualized therapies, once the personal molecular and genetic profile is known.

But the described biomedical model has been accompanied by inconsistencies. For the present purpose these will be briefly summarized by means of their respective anthropological, biological, and epistemological questions.

*(1) Anthropological Question.* The anthropological question is whether it really is possible to reduce the properties of a person simply to the molecular and genetic level. This seems to imply that the whole person is basically the result of his genotype and his molecular features, an assumption that is possibly not always intended but is the final logic behind the model. This includes a tendency to assume, at least tacitly, that a human being is determined ultimately by his genomic constituents and the molecular machinery, establishing not only his organic functions but also key aspects—if not all—of his personality. This is where many critics step in and require more flexible models that collide less with the human self-image as an autonomous person [14–26].

*(2) Biological Question.* Concerning the biological question, a somewhat paradoxical situation occurs: extensive empirical knowledge is moving increasingly beyond and above the paradigm that made this knowledge available. In many fields of study, it has become evident that molecular and genetic processes are subject to extremely refined regulation and control by the cell and the organism, so that it becomes difficult to define single molecular functions or certain genes as primary causes of specific processes. Rather, molecular functions and genes are integrated into a highly regulated network within the respective systems.

*(3) Epistemological Question.* The epistemological question revolves around the assumptions of whether an explanation can legitimately be derived from the underlying level of component parts and whether it will be possible at all to find primary causes for physiological and pathological functions [27–29].

The present paper will deal mainly with the second—the biological—question and present some evidence for the necessity to rethink the basic postulates about the organism and the corresponding research program described. There is no doubt that the program generated extensive achievements, but the results themselves show that a conceptual readjustment is necessary in order to understand the context in which the molecular and genetic processes are working.

This conclusion has been reached recently by researchers from quite different fields of biology and medicine [17, 22, 30–48]. Nurse [49], for example, formulated in* Nature *that biology now stands at an interesting juncture. The past decades have seen remarkable advances in understanding of how living organisms function at the molecular level, applying the idea that the gene is the fundamental unit of biological information and that chemistry provides effective mechanistic explanations of biological processes. At the same time, however, comprehensive understanding of many higher-level biological phenomena remains elusive. Even at the level of the cell, phenomena such as general cellular homeostasis and the maintenance of cell integrity are not fully understood. These gaps in knowledge are accompanied by a sense of unease in the biomedical community that understanding of human disease and therapy are progressing too slowly with respect to the expense for research funding. One reason for this, Nurse concludes, is that the past successes have led to an underestimate of the complexity of living organisms (see also [50, 51]).

Recent considerations of the evolutionary process, comparative embryology, epigenetics, and systems biology have begun to fundamentally change our understanding of organisms. In what follows, the results of some of these biological research areas will be summarized, and some anthropological and medical conclusions will be drawn.

## 3. Evolutionary Biology

The theory of evolution bears directly on human history and our relationships with each other and the world around us. Thus, in a very fundamental way it is tied up with ideas about human nature, including biological, social, and ethical issues. Against this backdrop it is interesting that especially the molecular studies of recent decades have led to an inconsistency that is beginning to change fundamentally the whole field of evolutionary biology. The central assumption of the “Synthetic Theory of Evolution,” which dominated the field during the second half of the twentieth century, was that the complete information on how to build an organism would be latent in the genes so that the genome provides a sort of blueprint that unfolds during development of the individual organism. The evolutionary process then would occur by random changes in the genome that would be selected and therefore yield benefits in survival and ultimately adaptation.

Contrary to this assumption, comparative studies have shown that, where most variation had been expected, in coding genes and in protein sequences, there is far-reaching conservation. Many genetic building blocks have undergone only minor or no changes over extremely long time periods, whereas organisms have changed significantly concerning morphology and ecology [35, 52, 53].

This conservation is valid for so-called housekeeping genes and developmental genes, which trigger the generation of organs and structures of the body during embryonic development, as well as in certain molecules such as globulins in hemoglobin or myoglobin. Such building blocks remained nearly unchanged over long evolutionary time periods. A major evolutionary transition, such as the Cambrian radiation (some 540 million years ago), during which the basic body-plans of all multicellular animals emerged, was a time of extensive innovations in metazoan morphologies and overall physiology, but not a time of larger innovations in cellular and molecular processes.

So where did the innovations for the major transitions come from? Comparative studies of recent organisms at different evolutionary levels increasingly show that it is mainly the* utilization* of genetic elements and of molecular processes that changed [52, 53]. Conserved genetic building blocks are used within new combinations, within a new context, or at different times or places during development. What has been changing during evolution is the regulation of genetic elements. This seems to be supported by extensive regulatory elements within the genome, which are highly responsive to influences from the surrounding system and the environment [54–56].

Thus, it is not possible to reduce evolution to an accumulation of mutations. If mutations do take place, in most cases these genetic building blocks are disturbed and consequently mistakes are generated. Possibly some crucial mutations might have taken place in regulatory regions of genetic elements or have generated larger genetic rearrangements, like duplications, for example, but at present it is not clear how they were generated.

Another riddle is that the same gene can be involved in the development of quite different organs. The same gene—in different situations or at different times of embryonic modifications—can be an element of completely different functions or organs. For example, genes that are involved in the generation of skeletal elements in early developmental stages of birds are again expressed during the generation of the beak (bone morphogenetic protein 4 (BMP4)). The famous Hox genes are not only involved in the anterior-posterior differentiation along the body axis, but also in the generation of limbs.

Questions and empirical results like these have triggered new thinking about biological evolution since the beginning of the new century (“extended evolutionary synthesis,” see also http://www.thethirdwayofevolution.com/) [53, 57–63].

## 4. Evo-Devo

An important young research area, which studies such processes, is the field of “Evolutionary Developmental Biology” (“Evo-Devo”). This approach conducts comparative studies of embryonic development from different evolutionary levels in order to see what changed over time in the respective developmental processes. The overarching idea is that developmental pathways must have changed to produce new types of organisms [53, 64, 65].

This field generated three fundamental insights as follows:

1. Developmental processes are always regulatory cascades: the expression of a gene leads to a protein that establishes a certain signal within the system, dependent on the state of the system. This signal—for example, a protein gradient or a rhythmic pulsation of a signaling factor—induces the expression of the next developmental gene, which is expressed in order to set off the next signal, and so on. Thus, embryonic development consists of cascades of developmental genes and their regulation within the system, generating interdependencies between both [66].
2. Even the very first processes within a fertilized egg cell follow this principle: within the cytoplasm there are crucial factors that determine the patterns of the first developmental steps and thus determine which genes are expressed in certain regions. The protein products of these genes then establish the next pattern of information.
3. Thus, there is no first cause: it is always the interdependence between systemic signals and genes (no gene, no next step; no signals from the system, no gene expression). The meaning or the role of an individual component within these cascades is dependent on its context [34, 67, 68]. Developmental genes are necessary at certain locations or times in the network, but they are no more important than other components of the system, as, for example, the morphological patterns that are already in place, providing positional information for the next genes to be expressed. It is also possible to express it this way: the system interprets the genome [69–71].
4. There is no such thing as a genetic program or plan of the organism or of its parts, such as a plan for legs or a head. The growing organism organizes itself through developmental cascades and uses different types of information. This information comes from positional information, gradients, and time structures such as rhythmic pulses, genetic, including epigenetic, information, and even environmental information [69, 72]. “Developmental Systems Theory” states that organi**s**mic form is constructed in developmental processes but not preformed in a genetic blueprint [39, 40, 73, 74].
5. In evolutionary transitions, these cascades have been modified so that developmental changes are an important source for innovations (“developmental plasticity”). The enormous variation of morphological form among animals, and ultimately even humans, is underlain by a common set of genes used differently in each systemic context, only appended by some changes, rearrangements, and supplements [63, 69, 70, 75]. This is in complete opposition to the assumptions of the architects of the Synthetic Theory, who viewed development to be irrelevant for the study of evolution [76]. Today it is acknowledged that developing organisms play a far more active, constructive role in both their own development and their evolution than the Modern Synthesis proclaimed [77, 78].

In these principles lies the answer to the riddle that the number of genes, which has been found in the human genome project, was far less than what had been expected for building the human organism and that the difference in the genomes of chimpanzees and humans has proven to be extremely small. What are the developmental rules by which two very different organisms are built?

It becomes increasingly evident that the information for this is found not only within the genome [79, 80]. Several other levels of information, such as chromatin-marking systems including methylation of cystines and histone modifications, alternative splicing, micro-RNAs, genomic imprinting, and many more, are involved [55]. Originally, chromatin marks were thought to be eliminated during transmission between generations. It is now clear that some of them can be transmitted across generations [81]. Such genomic marking may also underlie inherited maternal and nutritional effects and may be relevant for the inheritance of disease predispositions [59].

## 5. Epigenetics and Exploratory Systems

These epigenetic insights make it probable that organisms are able to deal much more flexibly with their genome than previously thought [55]. Epigenetic factors mediate developmental plasticity, the capacity of a single genotype to give rise to different phenotypes. This affects evolutionary dynamics by influencing the rate and direction of phenotypic change. Plasticity involves not just epigenetic changes in somatic cells and tissues; it can also involve changes in germline cells, which increases evolvability [81]. Shapiro [56, 61] stresses genomic changes in regulatory systems and speaks of a “read-write genome” as a much more adequate metaphor than the “read-only” genome, as genomic change often is an active cell-mediated physiological process.

In addition, during development many systems are generated by means of the function they begin to perform. Thus, the fine wiring of our brain is established by function. First a large amount of neuronal connections is established and only those connections will be retained that become functional during use. Such “exploratory systems” [52] are very flexible in order to serve the function they are taking over and during evolutionary changes.

Thus, it is not possible to reduce evolution merely to the genome. What changes during evolution are whole organisms, organs, physiological processes, developmental processes in embryos, and much more.

West-Eberhard [63] developed a theory according to which first the phenotype changed during evolution; the genetic elements would then follow. This is currently being discussed extensively as the phenomenon of “phenotypic plasticity” [57, 58, 80]. This again means that the phenotype has an ontological reality of its own, is not reducible to its genes, and is far more flexible than had been previously thought. For humans this means that we are much more actively involved in our own evolution by our behavior and culture.

From the viewpoint of an up-to-date theory of evolution, a much broader definition of inheritance is needed than was outlined by the Modern Synthesis. Badyaev [82] proposes that, in an empirical sense, inheritance is necessarily a combination of reliably transferred developmental resources needed to reconstruct, express, and modify genetically and epigenetically inherited components in a lineage. The statistical framework of quantitative genetics can sometimes distinguish among some of these components in terms of their transgenerational stability, also to identify genetic components of diseases, while in other cases this is much more difficult.

## 6. Increasing Autonomy in Evolution

As it becomes evident that the phenotype and the context of the system are much more relevant for evolution than has been thought up to now, patterns of phenotypic change are experiencing a renewed interest [83, 84]. One such contribution is the theory of increasing autonomy in evolution, which states that not only there are adaptations to a given environment, but also the generation of system levels during evolution leads to an increase in autonomy of the respective organisms. Especially during the major evolutionary transitions, organisms gained in stability, robustness, self-regulation, homeostasis, and flexibility. The direct influences of the environment were reduced gradually and a stabilization of self-referential, intrinsic functions within the respective systems was generated. In higher animals and in humans, this includes the potential for more flexible and self-determined behavior. These processes are described as changes in* relative* autonomy because numerous interconnections with the environment and dependencies upon it were retained [85, 86].

In humans, autonomy reaches a special level. First, there are those features we share with all mammals: a skin, which simultaneously tightly closes off the organism from the environment but is highly flexible and light; endothermy combined with a high aerobic capacity enabling movements with endurance and to a large extent emancipated from variations in ambient temperatures; an effectively stabilized fluid management, including refined processes for homeostasis, highly efficient renal functions, and an extremely refined immune system.

In addition, there are special features of humans that form the basis for the largely autonomous life that is cultivated today. Our hands are completely free from locomotor functions due to our upright posture and they have nearly unlimited possibilities of flexibility and dexterity. Humans are unique among primates in terms of relative brain size as well as some features of brain organization. One special feature of the human brain is that its prefrontal cortex is disproportionately large. It is involved in planning complex behavior, expressing personality, decision making, and moderating social behavior.

With all this, a feature emerged in humans that is clearly unparalleled within the animal world: the ability of self-control and willful, conscious behavior. The history of humankind must be told in terms of autonomy, increasing flexibility, and degrees of freedom.

There are many prerequisites necessary for all of these abilities, and our organism is constrained—but not determined—by many internal and external factors. But generally the organism provides a tremendously flexible and malleable basis for a human personality. Our autonomy is endangered if we have handicaps, if we are sick, if there are perilous influences, and if there are circumstances such as hunger or poverty, which prevent the unfolding of these capacities. Thus, here it is proposed that health should be defined in medicine and sociology as the capacity for a dynamically balanced autonomy and freedom of the individual.

## 7. Systems Biology

A further area of research to throw new light on the understanding of the organism is systems biology. Currently there are efforts, especially in molecular biology, to understand the abundance of detailed knowledge about organisms, which is available today, by a systems concept [38, 87–90].

Recent systems biology consists of two schools of thought which can be called “pragmatic systems biology” and “integrative systems biology” [91]. The majority of today's systems biologists belong to the pragmatic school, which studies large sets of molecular data in order to reconstruct systemic properties. In this context the term* system* covers a range of molecular interactions, which generate the whole function in question.

For integrative systems biologists, however, such an ad hoc approach is inadequate. For them it is crucial to analyze systems as systems and not as mere collections of parts in order to understand the emergent properties of compound interactions. Hence, systems are considered to constitute a fundamental ontological category.

**Figure 1:**
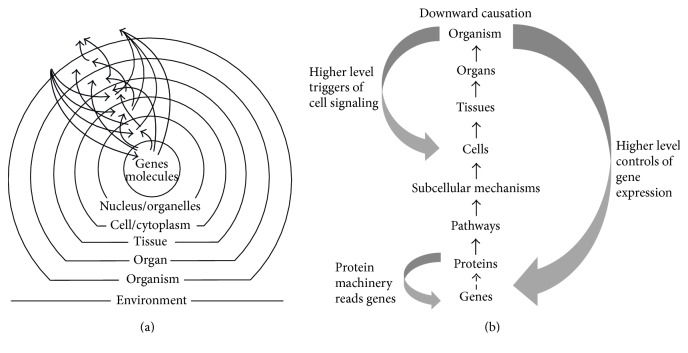
Schematic representations of the systems concept of Weiss [97] (a) slightly modified concerning the levels indicated and Noble [38] (b).

This second position is the older concept and much in accordance with recent empirical results. Beginning in the 1920s, Weiss was one of the earliest authors to introduce this systems aspect into biology [92–96]. Weiss defines a system as a relatively independent and stable entity [97]. Accordingly, a system generates restricting and regulative functions and imposes them upon its component parts, so that the functionality of the whole system is maintained. The system itself contains constituting properties and also information, which do not necessarily come from the components. At the same time each system depends on its components. The central illustration of Weiss is shown in Figure 1(a).

The cell is an example of such a system. It contains many components, but the system integrates them into a functional whole.

“…the basic characteristic of a system is its essential invariance beyond the much more variant flux and fluctuations of its elements or constituents” [97, p. 12].

This means that the components are not bound within determined processes, but rather can be variable, as is well known from cell biology today. The cell permanently regulates whether and when information is transcribed from DNA, whether certain proteins are generated, and which components are integrated into the membranes.

“This is exactly the opposite of a machine, in which the structure of the product depends crucially on strictly predefined operations of the parts. In the system, the structure of the whole determines the operation of the parts; in the machine, the operation of the parts determines the outcome” [97, p. 12].

Weiss further describes that a cell does not always work directly with its molecules but rather has subsystems, the organelles, which perform partial functions. Also the whole organism can be regarded as a system with several subsystems: the organs. Hierarchic, stepwise delegation of tasks to subsystems is nature's efficient instrument to allow an organism to keep order without having to deal with all its molecules directly.

This concept of Weiss is compatible with the more recent writings of Noble [38]. Noble describes how much progress has been made in dissecting systems into their smallest components. Now the challenge is to extend that knowledge up the scale. At each level of the organism, its various components are embedded in an integrated network of systems. Each such system has its own logic, so that it is not possible to understand that logic merely by investigating the properties of the system's components. The challenge is to learn more about the properties and conditions of each of these systems (see also [31, 39, 40, 98]).

In this sense the genome is just one level within this hierarchy and there is no reason to assume that the whole complex of integrated networks is determined by that level alone. Noble argues that we must look beyond the reductionist “gene's eye view” to answer the question of life. To understand what life is, we must make a radical switch of perception to view life at a variety of different levels, with interaction and feedback between genes, cells, organs, systems, bodies, and environments (Figure 1(b)).

## 8. The Concept of the Organism

In summary, recent results of evolutionary research, comparative embryology, and systems biology entail a conceptual shift in the way we think about organisms. Above, some of the components of such a concept have been discussed:

- The organism, like that of a human, is organized in different levels of systems.
- Each system level has level-specific rules that influence and regulate the components, which they contain and need for their proper functions.
- The different levels are interdependent and no single level can be privileged as a causal agency. DNA is just one level within this hierarchy.
- Evolution generated these system levels, so that the respective organisms gained in self-regulation, robustness, independence, and flexibility (“organismic autonomy”).
- Evolutionary changes occurred on different system levels and the phenotype played a decisive role (“phenotypic plasticity”).

A system is not a thing, but rather a process, which is organized during its individual development as well as during evolution and thus is not preformed in a physical or informational entity. Human beings are a result of an ongoing process of evolutionary integration and differentiation at several system levels [99].

These are quite recent considerations regarding the organism. There has been an older tradition of thinking in this direction, often summarized as “organismic biology” [30, 34, 100–103], but, only since modern analytical research has provided more detailed knowledge, it increasingly becomes possible to fill this approach with more content and evidence. In this sense it is a quite young research program (in the sense of Lakatos [104], see also [105]) and will need some time to develop in the future. Lakatos described the dynamics of research programs in general. In particular, successful ones, like the contemporary analytical approach, tend to dominate a field and can become quite resistant to major changes and conceptual progress. In this situation alternative approaches need protected niches as they may potentially become successful programs in the future.

## 9. Relevance to Health and Disease

These properties of the organism, gained from scientific observation, will gradually make it necessary to rethink the conceptual framework of physiology and pathophysiology in medicine [22, 59, 106–109]. When it becomes clear that genes are not causal agents, the popular assumption of the gene as a simple trigger of a certain trait and of diseases will be highly questionable [22, 35, 40, 56, 70, 110–112]. The idea that there is a gene for adventurousness, heart disease, obesity, religiosity, homosexuality, shyness, intelligence, or any other aspect of mind or body has no place any longer in the genetic discourse. Modern genetics is becoming much more systemic, and this will influence medical research profoundly.

Of course there are cases of simple monogenic disorders like sickle-cell anemia and cystic fibrosis. People with the defective genes always have the symptoms, whatever conditions of life they have. But this is rather the exception than the rule. They make up less than 2% of all the diseases that are known to have a genetic component and thus account for even much less of all diseases in general. For the remaining 98% of designated “genetic” disorders, the presence or absence of the disease and its severity are influenced by many genes and by the conditions in which a person develops and lives [35, 36]. Although the mutation of a single gene that greatly increases the probability of breast cancer is known, the large majority of breast cancer patients do not carry the mutation, a situation that is typical of so-called genetic predispositions to disease [113]. A monozygotic twin study showed that genomic sequence was a poor predictor of predisposition in 19 out of 24 common diseases examined [114].

As many people's understanding of the relation between genes and phenotype is based on the older simplistic view, there is a strong belief that biotechnology will give geneticists enormous power and that in the future they will be able to discover the cause and cure of most diseases. They will not only be able to read and translate the person's “book of life,” but also be able to edit the mistakes if necessary [35]. For cases of truly monogenetic diseases, it would be valuable if a genetic therapy would be available. For some of them it is now possible to test an embryo or a newborn and take steps for dealing with the situation quite early [115]. The success of imatinib for the treatment of chronic myeloid leukemia is another example. This, however, is not going to be the paradigm of diseases in general [113].

Single genes can penetrate quite differently. Classical genetics has known this for a long time and expressed it with the term “reaction norm” or more recently as “phenotypic plasticity” [63, 116]. In many cases, such systems are able to engage different pathways and functions in order to generate and maintain their overall integrity. This is well known in classical physiology and usually is described as “redundancy” [79, 80, 107, 117].

Noble et al. [59] reflect very critically on the role of the Modern Synthesis view of evolution as a driver of biomedical research priorities, experimental diagnostic, and therapeutic thinking since at least the beginning of the “War on Cancer,” in the 1970s. A key idea was that discrete genetic and molecular dysfunction led to specific cancer phenotypes. If these could be identified and then treated with drugs, cancer could be cured. Now this view is running into problems, and cancer is being regarded as a far more complex issue, involving many pathways, frequently triggered by environmental and/or behavioral factors [118, 119].

One consequence of the Synthesis view was the human genome project, which saw a tight linkage between genotype and phenotype, with two major outcomes envisioned [59]. For diseases with known genetic causes, cures based on gene therapy or other forms of genetic engineering would emerge. For more common noncommunicable diseases, such as diabetes and heart disease, common gene variants should explain much of the lifetime risk of the disease and lead to preventive medicine. Although scientifically it is valuable to have the knowledge from the human genome project now, it is nonetheless commonly agreed that the medical benefits have been disappointing. Understanding the functional context of the genome will be crucial for further progress [50, 120–124].

Noble et al. [59] argue that a more adequate consideration of the physiological perspective will improve the understanding of the genotype-phenotype relationship. It would bring the role of function to the study of evolution as well as medical topics, while it formerly has been excluded from examination, as just being the result of the actions of the genotype and other molecular processes. Function includes the higher order control that physiological systems assert over basic molecular processes. “Hormonal activity, metabolic networks or electrolyte regulation, to name but a few, represent physiological systems that are not restricted to specific gene activity, but affect the behaviour of numerous cells, tissues and developmental processes at once.” [59, p. 2241].

Classical physiology in general and integrative physiology specifically have been and still are studying integrative systems functions in the sense of “integrative systems biology.” Its task is to show how functions on different levels integrate lower-level functions and are themselves integrated into coherent systems. This demonstration will not emerge from lumping together large amounts of molecular data, based on which the higher-level processes could be calculated, as is the approach in pragmatic systems biology. Rather, it is necessary to develop further the methods that are appropriate to study functional systems on their own, including their integrative properties.

The principle of “developmental plasticity” is also highly relevant for medical questions, as the same gene in two humans may not result in the same phenotype, depending on many different factors such as environment, individual history, and epigenetic traits. This is but one component of the complexity, which is involved when geneticists try to predict traits or diseases in nonmonogenetic cases. Conversely, there can also be two or more networks of interactions, with different components, that end up producing identical phenotypes. Certain aspects of the phenotype seem to be remarkably invariant in spite of genetic and environmental differences. These are not new discoveries. Since the mid-20th century, geneticists realized that any character depends on a web of interactions between genes, their products, and the environment [35, 70, 116].

Jablonka and Lamb [35] describe how a vivid illustration of the intricacy and sophistication of genetic networks became apparent when geneticists started using genetic engineering techniques to disable (“knock out”) a particular gene and follow the consequences of this knockout on development. Much to their surprise, the scientists found that knocking out genes that were known to participate in important developmental pathways often made no difference whatsoever; the final phenotype remained unchanged. Somehow, the organism can compensate for the absence of a gene in some cases, using redundant pathways, but not in others [125].

Interestingly, in yeast 80% of the genes can be knocked out separately with little impact on survival in an unstressed environment [107, 126]. The key idea in physiology that a single mechanism frequently does not explain function and robustness of the system as a whole is shown perfectly by these results. In this case, the organism is able to recruit alternative pathways in order to maintain its autonomy.

The perspective of “physiological thinking” of Joyner and Pedersen [107] is much in accordance with the “integrative systems biology” approach described above (see also [31, 109]). From this perspective they state that targeting simple mechanisms for therapy may be insufficient in many cases; they describe that cardinal principles derived from physiology include the concepts of homeostasis, regulated systems, and redundancy.

“In this context, the most obvious explanation for the failure of molecular biology to deliver vast predictive or curative insights is perhaps general failure to understand the concept of redundancy operating in the context of homeostasis and regulated systems” [107].

Classical physiology has been discredited as being “merely descriptive” by reductionist thinkers, who promised to provide the “real causes” of organismic “mechanisms.” However, according to Joyner and Pedersen [107], these causes still remain to be shown.

Concerning the molecular viewpoint in general, it can easily be predicted that it will not be possible to develop something like molecular medicine, as has been widely propagated. There are, to be sure, molecular aspects of many diseases, and they might be important. But to reduce medicine simply to the molecular level will just not work.

According to Buchman [106] good health reflects the harmonious integration of molecules, cells, tissues, and organs and is dynamically stable. “At all levels—from genes to the web of organ systems that make up an individual—it is the balance of autonomy and connectedness that sustains health” (p. 246). Systems physiology suggests that healthy dynamic-stability arises by the properties of interconnected networks and attempts to learn how they can be influenced during therapy. Such regulatory medicine is a different approach than trying to influence single mechanisms. Different approaches may work in different situations, so these two concepts may not be exclusive.

Some of the medical disciplines that are presently summarized as “complementary medicine” try to influence this dynamic-stability, based on multilevel dynamics. In this sense they are predominantly a regulatory medicine and might deliver quite modern approaches to health and disease regarding the new insights of biology described above. So it should be interesting to consider their basic principles much more within scientific inquiry and to generate new types of research hypothesis from them [127–132].

In general, clinicians continue to rely most heavily on results from classical physiology, such as bedside haemodynamics and respiratory dynamics, to promote the autonomy and integrity of organismic functions, while science is dominated by a focus on simple tractable levels such as single cells or subcellular processes. Increasingly more authors therefore request to develop further approaches that are able to study the integrative functions that maintain autonomy [31, 106–109, 132].

## 10. Examples from Medicine

A few examples may show the relevance of the described systems view for medical research and practice.

### 10.1. Multiple Organ Dysfunction Syndrome

Buchman [106] presents an example from intensive care for integration versus disintegration of systems properties. Modern critical care is able to support or even temporarily replace physiological functions of vital organs, but this can run into multiple organ dysfunction syndrome (MODS), characterized by body-wide unbridled inflammation, remote in space and in time from the inciting event, leading to serial failure of several organs. It has been observed, as Buchman describes, that during the descent into MODS, physiological time signals (such as the beat-to-beat interval of the electrocardiogram) lose the fine variability observed in healthy patients. Greater regularity is thought to indicate increased system isolation. Thus, unbridled inflammation could cause uncoupling of organs from one another, thus precipitating MODS. It has been shown experimentally that the injection of bacterial endotoxin into human volunteers caused mild uncoupling, which manifests itself as loss of variability in the electrocardiogram signal. A similar uncoupling of autonomic regulation has been observed in patients descending into clinical septic shock and recoupling during the recovery period. The uncoupling phenomenon is also observed in the context of severe brain injury. Although such observations do not prove unequivocally that network disruption is the cause of MODS, the clinical imperative would seem to include restoration and protection of system integrity, and research needs to focus especially on such a phenomenon.

There are other examples for such an uncoupling of regulation, including recent findings that unusual meal times can induce a disruption of the circadian system. These results link energy regulation to the circadian clock at the behavioral, physiological, and molecular levels, emphasizing that the timing of food intake may have a significant role in obesity and thus might be relevant for medical and dietary intervention [133, 134].

### 10.2. Cancer

Another example of the contribution of a consequent systems thinking in medicine comes from oncology. While the field is largely dominated by the somatic mutation theory (SMT), which claims that cancer arises directly from a sequence of genetic changes in a cell that leads to its proliferation, there are some research groups posing alternative questions. For example, consideration has been given to whether the systemic context in which a tumor grows may be equally important, or even more important, than the genome of the tumor cells. Baker et al. [135–137] state that oncology today is facing “paradigm instability” concerning SMT due to paradoxical experimental results as well as the failure to solve the problem of the origin of cancer, so that such alternative theories might become quite important in the near future.

One such concept has been developed by Bissell [138–141]. She states that gene mutations are part of the process of cancer, but mutations alone are not always sufficient. Cancer involves an interaction between cells and surrounding tissue.

Thus, Bissell attempts to study breast tumors within their tissue environment. The basic idea is that cancer cells may turn into a lethal tumor with the cooperation of other cells and the extracellular matrix nearby. Genetic alterations are involved, but there seem to be reciprocal interactions with the surrounding tissue, and it remains to be shown which factors are actually the proximate cause. According to Bissell the microenvironment influences gene expression so that the behavior of a cell in normal tissues is largely determined by its interactions with the extracellular matrix, neighboring cells, and soluble local and systemic cues. Context and organ structure are essential in directing mammary gland development and differentiated function and in determining the response to oncogenetic insults, including mutations. She calls this concept “dynamic reciprocity.” Under this aspect Bissell presents an integrated view of development, cancer, and aging.

For many years, consideration of the tumor microenvironment had been ignored, but meanwhile it is getting more attention and also more funding [142–145].

Another concept has been developed by Soto and Sonnenschein [43, 135, 137, 146–150], proposing that cancers arise from a disruption of cell communication needed to maintain normal tissue architecture (tissue organization field theory, TOFT). The concept has two premises: (1) proliferation is the default state of all cells in a multicellular organism and the cells need functional integration and control within the system; and (2) carcinogenesis represents a problem of tissue organization comparable to the formation and development of organs. The central event during the generation of cancer is a loss of control over cell proliferation due to disruption of the intercellular signaling.

Meanwhile, some proponents of SMT extend the cellular view by a microenvironmental component and assume that the tumor microenvironment modifies the process by which mutations drive carcinogenesis, but with the primary focus on changes within cells. In contrast, TOFT takes a systemic, tissue-level view, where the disruption of cell communication and integration directly leads to cancer. Under TOFT, a byproduct of this disruption in cell communication is the appearance of mutations within tumor cells that might have only a minor or even no role in promoting carcinogenesis.

Both groups demonstrated that, in certain experimental settings, cells can become fatal tumors in one location and not in another. Baker [135] proposes several research topics, which are logical based on these alternative paradigms.

These concepts are offered here from a nononcologist viewpoint, but they are in accordance with the emerging view of the organism. One may wonder why some well-founded, though unconventional, approaches have been ignored for such a long time, regarding the urgent problem of cancer and the fact that the “war on cancer” has not been won, as Hanahan [121] formulates. Curiously, he still does not mention these alternative perspectives in his proposals for rethinking this war. The public is led to believe that science is following each clue to come to a solution for this disease. It appears, however, that paradigmatic obstacles hinder the development of a promising idea. This is true even though the paradigmatic nature of SMT was recognized more than thirty years ago [151]. Regarding the considerable funding of this research area, it should be possible to provide an alternative hypothesis an opportunity to see whether it may prove successful. Bissell had to struggle for 40 years to be heard and ultimately to get funding [152, 153]. Baker [135] shows elegantly how important it is to identify the theoretical framework of empirical research. Consequently, it becomes questionable why experimental scientists often are so reluctant to share openly their philosophical background. Broadly speaking, a shift towards systems-oriented approaches is necessary in cancer research [154].

### 10.3. Autism

Krimsky and Gruber [22] published a provocative book with the title* Genetic Explanations: Sense and Nonsense*. It includes some basic genetic discussions as well as several medical examples. One example concerns autism, for which Herbert [155] shows how the strong focus on the gene level can be a problem for clinical progress. For many years, autism has been considered a genetically hardwired neurodevelopmental disorder. If it all begins with genes, it seems clear that researchers need to find the genes that cause autism. It might then be possible to devise precise molecular or genetic interventions that would undo the damage or at least provide a way to control it. However, after decades and many millions of dollars devoted to looking for the genetic explanation of autism, there has been no home run. Instead, things have gotten even more complicated. Hundreds of genes have been identified, which may more or less contribute somehow to autism, while there has been little progress in therapeutics.

Herbert reports a whole series of anomalies undermining the genes-brain-behavior model, referring to more somatic and environmental factors with only minor genetic contributions, including possible dynamic changes in the condition. In her words, “evidence is shifting the conception of autism from a genetically determined, static, lifelong brain encephalopathy to a multiply determined dynamic systems disturbance with chronic impacts on both brain and body” (p. 129). Further integrative approaches have been proposed based on the assumption that autism might be associated with failure of the mirror neuron system [156–158].

Herbert proposes research efforts along a systems approach, including somatic, dietary, toxicological, environmental, and educational measures and therapies in order to manage the increasing autism rates. Yet, she complains that this approach is marginalized still, because it seems to be implausible in a simple gene-brain-behavior narrative and that this disregard has had severe practical consequences.

In the same volume Joseph and Ratner [159] show how fruitless the search for genetic causes in psychiatry and psychology in general has been, although reports appear regularly on genetic causes for diseases like depression, bipolar disorder, or schizophrenia. The same holds true for personality traits and cognitive abilities. They state that the field has published thousands of candidate gene association studies but few if any could be replicated. Also the general “wisdom” of genetic vulnerability and environmental exposure has not generated any direct evidence. They state that the fields of behavioral genetics and psychiatric genetics are rapidly approaching a period of crisis and reexamination (for another discussion of these problems see chapter 11: “Genes and disease,” within the excellent book of Parrington [55]).

## 11. Integrative Systems Biology in Medicine

According to the multilevel integrative systems theory, there can be many levels on which malfunctions may become manifest. One such level might be the genetic or the molecular level, but in other cases more integrative levels might be involved and might be the primary target of medical intervention. In this sense, McEwen and Getz [160] advocate for an open and far-reaching definition of personalized medicine, rather than restricting it to high-tech medicine based on biomolecular mapping and tailoring of drug therapies. A similar approach for chronic, noncommunicable diseases is proposed by Bousquet et al. [161]. In this sense there are many reservations about the present focus of personalized medicine and about its potential to fundamentally change medical practice, although it is agreed that it will widen the portfolio of tools that medicine can use [12, 15, 18, 19, 24, 113, 162]. On the other hand several complementary medical approaches are based on integrative concepts of the organism and thus might come much closer to a personalized medicine [127–129, 132].

André et al. [163] wonder why, despite outstanding results in lung cancer, colorectal cancer, and melanoma, only a few predictive biomarkers are currently justified in routine clinical practice. They see a persistent gap between the growing number of identified deregulated pathways or genetic mutations and their actual implementation at the bedside as part of clinical routine. The readjustment of organismic thinking, which is proposed in the present paper, might help to find out why. One might even speculate that the relevance of biomarkers in general will more consistently be available when the system context is known and considered [164]. In the same sense, Roden [165] asks for a solution to the “genotype-phenotype dilemma” in clinical decisions.

A more realistic, multilevel understanding of the organism might also readjust research funding and politics. Thus, thinking about the organism has profound consequences and is not just an intellectual exercise.

The clinician must include more aspects than just the biological ones. Kohane [162], from Harvard Medical School, argues that personalized medicine, as currently understood, “at best provides an incomplete model of the patient and at worst can lead to grossly inappropriate practices.” Personalization of medicine, he formulates, requires a well-grounded understanding of who the patient is, an understanding of the subpopulation that most resembles that patient in the context of the decisions, the environment, and the physiology. Molecular measurements are only one of many clinical characterizations, and often not the most important one, according to Kohane.

Vogt et al. [166] state that integrative systems biology can contribute to rehabilitating “the person” in human biology, thus building a much more adequate background for humanistic medicine. They conclude, however, that it still does not integrate central qualities of a person such as consciousness, meaning, and value. This is correct, as integrative systems biology only attempts to come to a more coherent notion of the living organism (the level of “being alive”), identifying the instrument for psychical and cognitive abilities in humans.

The proposal in the present paper, to merge systems biology with the autonomy approach and the concept of phenotypic plasticity, at least provides an understanding of the flexibility and malleability of the organism, which is necessary for an autonomous, self-determined person. This is how the approach may contribute to disband the conflicting views of humanistic and biomedical concepts without contradicting the results of empirical research.

## 12. Conclusion

Some areas of biology are currently experiencing a conceptual shift in the way we think about organisms. The hallmark of such a reform is a relinquishment of any privileged levels of causation in physiological inquiry. This will also influence medicine, which needs an enhanced notion of the organism as a multilevel, highly flexible, adaptable, and, in this sense, autonomous basis for a human individual [167]. Medical physiology and pathophysiology need to become more organismic and less reductionistic. A further elaboration of integrative systems physiology, as described above, might stand at the core of these developments.

Health can be described in medicine and psychology as the capacity for dynamically balanced systems autonomy. All physiological functions serve the establishment and maintenance of this autonomy: the highly regulated functions of endothermy, circulation and blood pressure, electrolyte balance, exchange of respiratory gases and nutrients, neuronal regulation, functions of the skin, and many more. It is the daily work of a physician to adjust the functional autonomy of systems when they become out of balance and limit the autonomy of the patient.

Some of these systems work similarly in different patients, so that standard therapies can be applied, whereas there can be extensive individual variations in other systems, according to different personal histories, lifestyles, time patterns, and personalities. And every patient has his or her own form of a more or less stabilized personal autonomy and his or her own technique to maintain it or to fail. In this sense autonomy, integrative systems biology, and phenotypic plasticity need to be central notions for the development of “personalized” medicine.

Humans establish personal and psychological autonomy, which is described and applied in psychology as “resilience” in recent years [15, 168, 169]. Resilience is the stability and robustness of individuals being able to interact in a positive way with their environments. They can effectively navigate their way around crises and utilize effective methods of coping. This is the continuation of the biological concepts described here to the psychological level.

Many techniques may need to be developed in order to describe and study system functions and how their multilevel dynamics will affect the entire system, but there may be a large potential for medicine to develop explicit strategies to influence these dynamics.
